# How Can Physical Activity Be Promoted Among Children and Adolescents? A Systematic Review of Reviews Across Settings

**DOI:** 10.3389/fpubh.2019.00055

**Published:** 2019-03-19

**Authors:** Sven Messing, Alfred Rütten, Karim Abu-Omar, Ulrike Ungerer-Röhrich, Lee Goodwin, Ionuţ Burlacu, Günther Gediga

**Affiliations:** ^1^Department of Sport Science and Sport, Friedrich-Alexander University Erlangen-Nürnberg (FAU), Erlangen, Germany; ^2^Department of Sport Science, University of Bayreuth, Bayreuth, Germany; ^3^Department of Psychology, University of Münster, Münster, Germany

**Keywords:** physical activity, children, adolescents, review, family, childcare, family, recommendations

## Abstract

**Introduction:** A vast majority of children and adolescents are physically inactive. As a result, high obesity rates and related diseases have made physical activity promotion a politically relevant topic. In order to form the basis for political decision making, evidence is required regarding the efficacy and effectiveness of interventions for physical activity promotion. In contrast to previous research, this systematic review of reviews targets three key settings (family and home, childcare, school), and is among the largest to have been conducted.

**Methods:** A systematic review of reviews was conducted as part of a large-scale project to develop national recommendations for physical activity promotion in Germany. Six electronic databases were searched and inclusion criteria were defined. Two independent reviewers screened the titles and abstracts of potentially relevant literature. 213 reviews were identified and categorised by target group. A total of 74 reviews were identified dealing with children and adolescents. Each review underwent a quality assessment.

**Results:** 39 reviews with the highest quality and relevance were analysed. Three reviews focused on the family and home setting, 4 on the childcare setting, 28 on the school setting and 4 on other settings. Evidence revealed the key role played by parents in promoting physical activity in children within each setting. Furthermore, evidence pointed toward the efficacy of multi-component interventions in the childcare and school setting. Several evidence-based intervention strategies were identified for childcare facilities and schools.

**Discussion:** The review of reviews identified a number of promising strategies for PA promotion among children and adolescents. Among reviews, multi-component interventions in childcare facilities and schools stand out prominently. At the same time, the review of reviews indicated that there is still a lack of studies on the efficacy of interventions that go beyond the individual level. We recommend that future research should also target community and policy level interventions and interventions other than the school setting. In order to make more specific recommendations regarding the scale-up of promising intervention strategies, further knowledge about the effectiveness, health equity and cost effectiveness of interventions is needed.

## Introduction

With recent studies showing that 124 million children worldwide are obese (1), physical activity (PA)—alongside nutrition—is a key in the fight against childhood obesity. Nevertheless, 75% of boys and 85% of girls in Europe (2) do not fulfil the World Health Organisation (WHO) recommendations of at least 60 min of moderate-to-vigorous-intensity PA per day (3). As the impact of PA on children's physical, psychosocial, and intellectual development is well proven (4), PA promotion is a highly relevant topic.

On the political level, central organisations are calling for action in order to combat childhood obesity. In 2011, the General Assembly of the United Nations declared that non-communicable diseases are one of the largest challenges of the twenty-first century, and that all sectors need to generate effective responses for the prevention and control of non-communicable diseases (5). Additionally, the European Union published an Action Plan on Childhood Obesity 2014–2020 (6), and the WHO formulated specific recommendations in their report “Ending Childhood Obesity” (7). Furthermore, efforts specifically focusing on PA promotion are increasing. For example, the European Union Physical Activity Guidelines recommended a number of policy actions (8), while the WHO developed a European Physical Activity Strategy for 2016–2025 (9).

Due to the high relevance of this topic in politics and public health, a large number of reviews have been conducted to identify effective interventions for PA promotion (10). From the perspective of evidence-based medicine, the preferred method of knowledge summarisation is through the conduction of a systematic review of reviews (11). Such evidence is needed as the basis for decision making and allows research to have an impact on policy and practice (12).

Over the past years, interventions for PA promotion have been investigated in reviews of reviews. Steenbock et al. (13) analysed the efficacy of interventions to promote PA and healthy eating in the childcare setting, while Kriemler et al. (14) dealt with the effects of school-based interventions on PA and fitness in children and adolescents. Moreover, the overarching review of Heath et al. (15), which investigates many different types of PA interventions (not only for children and adolescents), also includes findings on school-based interventions.

In contrast to the above reviews, our systematic review of reviews summarises the best available evidence for the target group of children and adolescents across the settings of family and home, childcare and school. Such information is required for the drafting of policy recommendations on the use of intervention strategies in settings to promote PA among children and adolescents. Another reason for obtaining such information is to identify potential “best buys” for PA promotion. Additionally, our review of reviews across settings was conducted to shed light on potential research gaps. The results were not only utilised to synthesise evidence, but have also formed parts of the German recommendations for PA promotion. To our knowledge, this review of reviews is the largest to be conducted with analysis on the efficacy and effectiveness of PA promoting interventions for children and adolescents.

## Methods

This systematic review of reviews was part of a large-scale project to develop national recommendations for PA and PA promotion. The overarching aim was to provide recommendations by target group: children and adolescents, adults, older adults, adults with a chronic disease, general population. This article provides an in-depth overview of results for the target group “children and adolescents.”

In order to formulate recommendations that centre on the efficacy and effectiveness of interventions, a systematic review of reviews was conducted. Six electronic databases (PubMed, Scopus, Sport Discus, PsycInfo, ERIC, IBSS) were searched in 2015 for all publication years using the following search terms: “physical activity,” “intervention,” “evidence,” “effect,” “health,” and “review.” Alternative terms (e.g., bike, biking, cycling, walking, active transport, human powered transport, sedentary, exercise, sport) were defined and MESH-terms were formulated. Once the relevant literature had been identified, titles and abstracts were screened by two independent reviewers. The screening process was based on the following criteria: (a) The review contains empirical results from single studies. (b) The review includes interventions centred on the promotion of PA or the reduction of inactivity. (c) The review focuses on the efficacy and/or effectiveness of interventions. (d) Some of the single studies included in the review are of longitudinal design. (e) Reviews were written in English or German. Duplicates were excluded.

The titles and abstracts of the identified records were screened to ensure that the above inclusion criteria were met. Two reviewers independently screened full texts from 223 reviews in a secondary screening process. Additional hand searches were conducted to identify further reviews. The remaining 213 reviews were then categorised by target group (children and adolescents, adults, older people, people with preconditions, general population).

**Figure 1:**
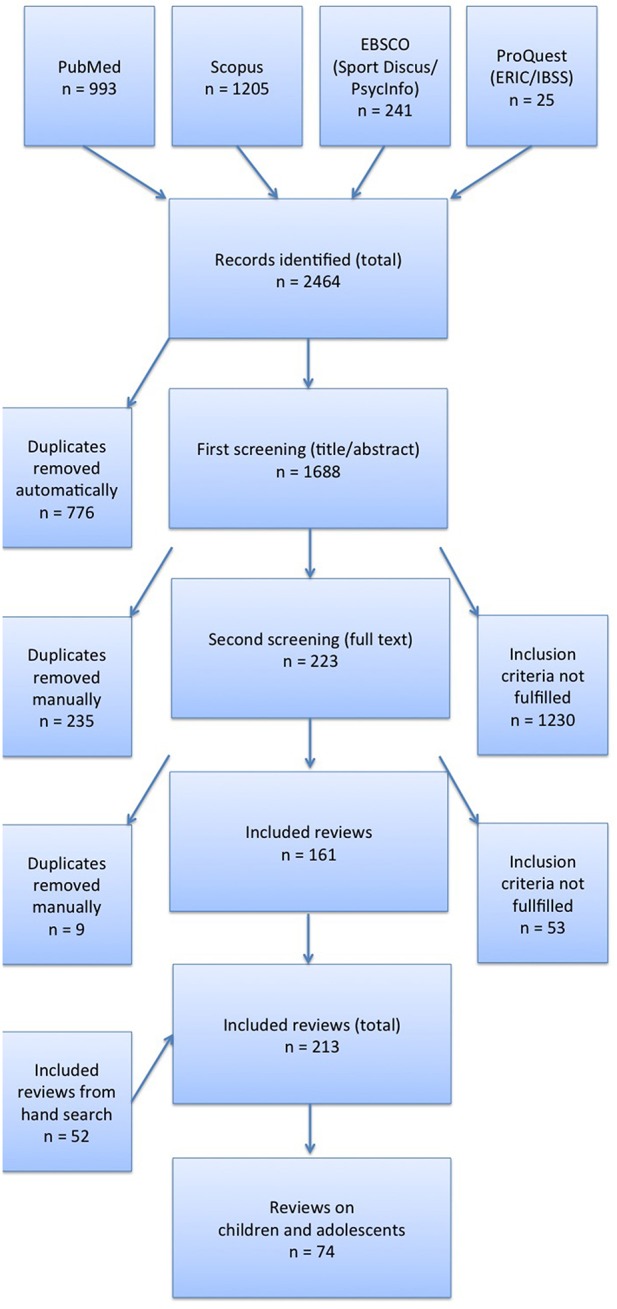
Flow chart.

Seventy-four reviews dealt with the target group of children and adolescents (see Figure 1). Out of the 74 reviews, 51 were identified as a part of this systematic review of reviews, and 23 were obtained via hand search. Each of the 74 reviews underwent analysis. During the process of formulating national recommendations for PA, reviews were excluded due to the following reasons: reviews did not fulfil the inclusion criteria (16–21), did not deal with intervention studies (22), did not focus primarily on PA (23–30), were conducted unsystematically (31–39), only included a few studies dealing with PA promotion (40–42), were outdated or reported limited results (43–47), or were of very low quality (48). Once the exclusion process was complete, the total number of remaining reviews consisted of 39.

One independent researcher assessed the quality of reviews using the AGREE instrument (49). This instrument was utilised in the formulation of the Canadian Physical Activity Guidelines (50).

The AGREE instrument comprises of five criteria that deal with the methodological quality of studies. However, as the AGREE criteria are relatively easy to fulfil, methodologically weak reviews also receive high ratings. To counter this issue, an additional instrument was developed to enhance the accurate assessment of methodological quality for the identified reviews and meta-analyses (see Table 1). In order to assess review quality, both instruments were applied by calculating percentage values for each review. These values showed the percentages of fulfilled criteria per review, both on the basis of the AGREE criteria and the criteria from our newly developed instrument. The percentage values recorded for each review were calculated based on applicable criteria (e.g., some criteria were only applicable for meta-analyses).

**Table 1:** Quality checklist.

**Criterium**	**Explanation**	**Meta-analysis**	**Review**
Search terms and links of the search terms are stated explicitly (51).	Without this information the study is not transparent.	Yes	Yes
It is stated which databases were searched (51).	Without this information the study is not transparent.	Yes	Yes
There is a diagram for included/excluded studies (51).	This is considered as standard.	Yes	Yes
The included studies are shown in tables (min. design, measures, outcomes) (51).	This is considered as standard.	Yes	Yes
There are estimations of the size/effects of different bias factors (51).	This is considered as standard.	Yes	Yes
The problem of dependent measurements in the aggregation is discussed or dealt with (by exclusion or by statistical treatment of the dependencies) (52, 53).	Dependent measurements in studies lead to an overestimation of effects. As a minimum, this problem should be addressed. Methods for aggregating dependent measurements are on the market but are not used often.	Yes	When appropriate
Effect sizes and not only ordinal assessments of primary study results are reported (53).	For meta-analyses inacceptable. However, for many reviews an averaged effect size can be reported (but not always).	Yes	When appropriate
Furthermore, (only or mainly) effect sizes without a statistical bias are reported (Hedges'g or log-OR) (53).	Changes in percentage values show—depending on the baseline value—a bias. Because of that, summaries of unbiased mean values are preferable. For reviews, this depends on the data availability.	Yes	When appropriate
More than 5 primary studies per analysis are reported (except in subgroup-analyses, see below) (53).	For 5 or less studies a summary depends strongly on the single study. These reviews/meta-analysis are less useful.	Yes	Yes
An analysis of the publication bias was conducted (e.g., funnel plot or variance analyses) (53, 54).	This is a standard for the estimation of the publication bias in meta-analyses.	Yes	No
Forest plots are reported (53).	This is a standard in meta-analyses. In reviews forest plots should be reported when appropriate data are available.	Yes	When appropriate
A check of the study heterogeneity was conducted (l-square and *p*-value) (53).	This is a standard in meta-analyses. In reviews this should be discussed at least regarding the existence of heterogeneity.	Yes	When appropriate
Heterogeneous results are not only reported, but also discussed (53).	This is a standard in meta-analyses. In reviews this should be discussed at least regarding the existence of heterogeneity.	Conditionally (l12 = 1)	When appropriate
For clarifying heterogeneous results, meta-regressions, or subgroup-analyses are conducted (53).	This is a standard in meta-analyses. Not applicable for reviews.	Conditionally (l12 = 1)	When appropriate
It is evident that the results are/were not only caused by one/a few big study/-ies (53).	This should be checked both in meta-analyses and in reviews.	Yes	Yes
Very small but significant effects (|g| < 0,10; |LOR| < 0,10) are discussed regarding their relevance (51).	Such results are possible in meta-analyses. It is a problem that both model violations and dominant studies result in such effects. A discussion is essential. Only reporting “significances” is not helpful.	Yes	No
Insignificant or very small but heterogeneous effects are analysed by using sensitivity analyses, meta-regressions or subgroup-analyses (51).	This should be done in meta-analyses.	Yes	No

Combining the results of AGREE and our instrument ensured reliability when differentiating between the quality of reviews (especially for high quality reviews), thus improving the overall quality of assessment. Based on the combined results obtained from both instruments, the quality of each review was defined as high, medium or low.

Following the methodology proposed by Smith et al. (11), reviews were evaluated independently by two researchers. The evaluation focused on summarising evidence pertaining to various intervention types.

A narrative synthesis of these 39 reviews is presented in this manuscript. The synthesis presents the results regarding the efficacy and effectiveness of PA promoting interventions. While efficacy trials test whether an intervention works under optimum conditions, effectiveness trials test an intervention under real-world conditions (55). However, even though the distinction of efficacy and effectiveness is highly relevant in health promotion research (56), both terms are often used interchangeably. When summarizing the main findings of the authors in our tables we overtook their wording, so that it might seem that many reviews analysed the effectiveness of interventions. In reality, and as stated in the discussion, most reviews analysed their efficacy.

## Results

### Overview

Table 2 provides an overview of the quality of reviews analysed in different settings.

**Table 2:** Quality rating of the included reviews.

**Setting**	**Number of reviews**	**Type of review**	**Mean level of quality (QC) (%)**	**Mean level of quality (AGREE) (%)**	**High quality**	**Medium quality**	**Low quality**
Family and home setting	3	3 systematic reviews	74.0	72.0	2	1	–
Childcare facilities	4	1 reviews of reviews, 2 systematic reviews, 1 unsystematic review	68.0	76.3	3	–	1
School	28	2 reviews of reviews, 25 systematic reviews (3 of them with meta-analysis), 1 unsystematic review	64.2^a^	77.4	14	9	4
Other	4	4 systematic reviews (2 of them with meta-analysis)	67.8	80.3	3	–	1

a *In the school setting, the quality rating included 27 of the 28 reviews. To one of the reviews the quality rating was not applicable as the consensus method was used*.

### Family and Home Setting

Three systematic reviews were identified that deal specifically with interventions in the family and home setting (Table 3) (57–59).

**Table 3:** Reviews focusing on the family and home setting.

**References**	**Type**	**Number of included studies**	**Quality rating (QC/AGREE)**	**Intervention type**	**Interventions included in review**	**Main findings of the authors**
**FAMILY AND HOME**						
Marsh et al. (57)	Systematic review	17	Medium (75%/69%)	Informational, behavioural and social	Multiple family-based interventions in different settings	14/17 studies reported sig. decreases in sedentary behaviour. Evidence for effectiveness of interventions that involve parents in a medium-to-high intensity.
Mitchell et al. (58)	Systematic review	20	High (70%/75%)	Identification of correlates of children's PA (no intervention)	–	Positive associations were found between children's PA levels and the following variables: Parental PA level (8/10 studies), parental encouragement (2/6 studies), instrumental parent behaviour, and parental support (3/4 studies).
Xu et al. (59)	Systematic review	30	High (77%/72%)	Identification of correlates of children's PA (no intervention)	–	8/11 studies reported positive associations of parents supporting children's PA. 8/10 studies reported positive associations of parent role modelling (parents' PA) with children's PA. Some parental influences are significantly associated with young children's PA with moderate to strong evidence.

All three reviews stated that parental encouragement and support can increase PA in children (58, 59), as well as reduce sedentary behaviour (57). Two reviews showed that parental behaviour influences the behaviour of their child. While one review identified a positive association between children's PA levels and the PA levels of their parents (58), another review found that a reduction in parental (sedentary) screen time can lead to decreased screen time in children (59). One review concluded that the level of parental involvement is more important than the setting in which the intervention is conducted (57).

The availability of PA equipment seemed to have a positive effect on children's PA levels, whereas busy work schedules and parent fatigue demonstrated negative effects (58).

Inconsistencies were found regarding the influence of parental enjoyment, maternal depressive symptoms, maternal self-efficacy, parental rules limiting sedentary behaviour, parental perceptions on the importance of PA, the level of child safety, and the child's physical competence (58).

### Childcare Facilities

Four of the identified reviews dealt with PA promotion in childcare facilities (Table 4). In general, the majority of interventions for pre-schoolers seem to be centre-based and teacher-delivered (60).

**Table 4:** Reviews focusing on the childcare setting.

**References**	**Type**	**Number of included studies**	**Quality rating (QC/AGREE)**	**Intervention type**	**Interventions included in review**	**Main findings of the authors**
**CHILDCARE FACILITIES**						
Ling et al. (60)	Systematic review	23	High (88%/82%)	All	Multiple interventions	8/20 interventions resulted in sig. positive changes in objectively measured PA. All 8 sig. interventions were centre-based and included a structured PA component, 6 included multiple components, 5 integrated theories or models, and 4 actively involved parents.
Mehtälä et al. (61)	Systematic review	23	High (69%/83%)	Behavioural and social, policy, and environmental	Structured PA, playground or playtime modifications, teacher and parental involvement	14/23 studies reported sig. positive changes in children's PA levels. The high quality studies were more likely to report a significant increase in children's PA than the lower quality studies, except for playground or playtime modifications.
Steenbock et al. (13)	Review of reviews	5	High (61%/72%)	Behavioural and social, policy, and environmental	PA programs, increase of time for unstructured PA, teacher training	2/5 studies report positive changes in moderate to intensive PA. Effective interventions consisted of teacher training and a daily PA program.
Ward et al. (62)	Unsystematic review	8	Low (54%/68%)	Behavioural and social, policy, and environmental	PA programs, increase of time for unstructured PA, playground equipment, teacher training	2/5 studies investigating PA programs resulted in sig. positive changes in children's PA levels. 2/3 studies investigating environmental and policy interventions resulted in sig. positive changes in children's PA level (teacher training, playground equipment). Regular structured PA programs can increase the amount and intensity of PA that children receive.

The reviews stated that theory or model-based interventions are more successful. The review by Ling et al. (60) stressed that most theory or model-based interventions show a positive effect. In contrast, only one third of non-theory-based interventions were effective. The authors also indicated that the use of appropriate behavioural change strategies may be more important than theoretical models (60).

Furthermore, based on the available evidence, Ling et al. (60) concluded that multi-component interventions are more successful than single-component interventions. One of these components should be a structured PA programme (60, 62). Interventions that had a positive effect on PA behaviour in children integrated structured PA into the formal curriculum (60, 62). Both reviews stressed that the increase of structured PA should not be implemented at the expense of children's play time (60, 62).

Additionally, as children are most active during the first 10–15 min of being outdoors, experts suggest the provision of more free time (62). Another way to alter the free play environment at preschool is to provide additional play equipment—when play equipment isn't provided, children are more prone to sedentary behaviour and engagement in games that promote inactivity (62).

To conduct interventions for PA promotion, Mehtälä et al. (61) recommended PA-specific in-service teacher training. Both experience and personal characteristics played an important role in the promotion of PA among children in the childcare setting (61). Furthermore, Ward et al. (62) stated that there is a need for teacher training, and that structured PA programs should be implemented by staff who are specialised and well-trained. Having well-qualified staff could also support skill development and competence in children (13).

Two reviews recommended involving parents (13, 60), while one review stated that evidence on parental support “remains unclear” (61). Nevertheless, Mehtälä et al. (61) also stated that family is the most influential setting for young children, and that a partnership between families and childcare is crucial. Regarding specific strategies for parental involvement, providing health promotion information to parents is recommended in one review (13).

### School

28 of the identified reviews focused on the school setting (see Table 5). Strong evidence indicates that multi-component interventions are effective in the promotion of PA (14, 63–69). While multi-component interventions are more effective than single-component interventions (14, 70) they do not seem to produce synergistic results (63, 71).

**Table 5:** Reviews focusing on the school setting.

**References**	**Type**	**Number of included studies**	**Quality rating (QC/AGREE)**	**Intervention type**	**Interventions included in review**	**Main findings of the authors**
**SCHOOL**						
Atkin et al. (80)	Systematic review	9	High (71%/72%)	Behavioural and social	After-school programs	3/9 studies reported sig. positive changes in PA with small effect sizes. No evidence for effectiveness. Potentially more effective when interventions target PA alone.
Barr-Anderson et al. (79)	Systematic review	23	High (83%/73%)	Behavioural and social	Short PA breaks	12/15 studies reported sig. positive changes in PA with moderate to large effect sizes. Short PA breaks are a promising strategy to increase PA.
Beets et al (81)	Systematic review with meta-analysis	13	Medium (35%/73%)	Behavioural and social	After-school programs	Combined effect size from 6 studies is 0.44 for increasing PA. After school programs can increase PA.
Brennan et al. (72)	Systematic review	24 interventions	Medium (55%/78%)	Policy and environmental. Behavioural and social	School PA policies and environmental strategies. Active transport	Recommended: School PA policies and environments. Promising: Ensuring safe routes to school.
Broekhuizen et al. (82)	Systematic review	13 experimental studies 17 observational studies	Medium (71%/65%)	Policy and environmental	Change of school environment	Moderate evidence for the provision of playground equipment on increases in PA. Inconclusive evidence for playground markings, allocating play space, multicomponent interventions. No evidence for decreasing playground density, PA promotion by staff, increasing recess duration.
Chillón et al. (86)	Systematic review	14	Medium (55%/91%)	Behavioural and social	Active transport	12/14 studies reported increases in active transport. Effect sizes: 2 trivial, 5 small, 2 large, 1 very large. Interventions show small but promising effectiveness in increasing active transport.
Crutzen et al. (63)	Systematic review	20	High (71%/91%)	All	Multiple interventions	13/20 studies reported sig. effect on PA. Interventions that included environmental components resulted in larger effect sizes. Interventions aimed at multiple behaviours resulted in smaller effect sizes.
De Meester et al. (71)	Systematic review	20	High (62%/85%)	All	Multiple interventions	13/20 studies reported sig. effect on PA. Majority of interventions lead to short-term improvements in PA.
Dobbins et al. (77)	Systematic review	26	High (85%/82%)	All	Multiple interventions	3/6 studies reported sig. increase in PA rates. 5/7 studies reported sig. increase in time spent in PA. Good evidence that interventions increase duration in PA.
Dobbins et al. (78)	Systematic review [update of Dobbins et al. (77)]	44	High (85%/82%)	All	Multiple interventions	2/5 studies reported sig. increase in PA rates. 12/17 studies reported sig. increase in time spent in PA. Effect sizes are generally small. Some evidence that interventions are effective in increasing PA.
Dudley et al. (76)	Systematic review	23	Medium (57%/86%)	Behavioural and social	Physical Education lessons	13/19 studies reported sig. increase of participation in physical education lessons. Most effective strategies: Direct physical education instruction and supporting the professional development of teachers.
Escalante et al. (83)	Systematic review	8	High (72%/73%)	Policy and environmental	Change of school environment	Interventions on playground markings plus physical structures increase PA in schoolchildren during recess (short to medium term).
Heath et al. (15)	Review of reviews	5	High (80%/82%)	All, but effectiveness is only shown for behavioural and social	Multiple interventions	Effectiveness of school-based strategies encompassing physical education, classroom activities, after-school sports, and active transport.
Kriemler et al. (14)	Review of reviews + updated review	4 + 1	High (66%/82%)	All	Multiple interventions	Review of reviews: Multicomponent interventions in the school setting are the most promising strategy. Updated review: 16/16 studies reported sig. increases on at least one PA dimension.
Langford et al. (64)	Systematic review	18	High (93%/92%)	Policy and environmental	School curriculum and changes of the school environment	Pooled effect size of 6 studies is 0.14 for increase in PA. Interventions were able to increase PA levels.
Larouche et al. (87)	Systematic review	68	Medium (60%/77%)	Behavioural and social	Active transport	22/28 studies reported positive associations between active transport and PA. The majority of studies shows the effectiveness of interventions for active transport.
Lonsdale et al. (75)	Systematic review with meta-analysis	14	High (80%/82%)	Behavioural and social	Physical education lessons	8/13 studies reported sig. increases in time spent in moderate/vigorous PA. Overall, interventions led to a 10% increase in time spent active during physical education lessons across studies. Evidence for the effectiveness of interventions in increasing PA time during physical education lessons.
Naylor et al. (84)	Systematic review	18	High (70%/82%)	All	Multiple interventions, implementation	11/15 studies reported a positive relationship between implementation and at least one health outcome.
Parrish et al. (67)	Systematic review	9	Low (50%/60%)	Behavioural and social	After-school programs and active breaks.	5/9 studies reported a positive effect on PA during recess. Inconclusive but promising evidence for the effectiveness of school recess interventions on PA.
Pate et al. (73)	Systematic review	43	High (66%/82%)	Policy and environmental	Policies to increase PA in children and adolescents	Strong evidence for effectiveness: Policies for increasing physical education and improving school environment. Moderate evidence: Active transport. Limited evidence: PA related health education.
Public Health England (69)	Review and expert consensus	Unclear	Quality rating not applicable (expert consensus)	All	Multiple interventions	Evidence rating 3/5 for effectiveness of multi-component interventions. creating active environments, and promoting active travel.
Quitério et al. (68)	Systematic review	27	Low (30%/60%)	Behavioural and social	Physical education lessons	A considerable amount of physical education interventions improved self-reported and objectively measured PA, student activity levels during physical education lessons, physical fitness and other health-related outcomes.
Salmon et al. (70)	Systematic review	57	Low (50%/65%)	All	Multiple interventions	Effective school setting interventions included focus on physical education, activity breaks, and family strategies.
Van Grieken et al. (88)	Systematic review with meta-analysis	34	Medium (58%/86%)	Behavioural and social	Reducing sedentary behaviour	13/34 studies reported sig. decrease in sedentary behaviour. Compared to control groups the reduction was 20 min/day in average. Interventions in the school setting to reduce sedentary behaviour can result in significant decreases in sedentary behaviour.
Van Lippevelde et al. (85)	Systematic review	5	Low (22%/68%)	All	Parental involvement in school interventions	Inconsistent evidence that parental involvement is important to improve the effectiveness of school-based interventions.
Van Sluijs et al. (65)	Systematic review	57	High (80%/74%)	All	Multiple interventions	Out of 14 studies: Strong evidence that school interventions with family or community involvement can increase PA. Out of 13 studies: Inconclusive evidence that school interventions with no community/family involvement are effective.
Waters et al. (74)	Systematic review with meta-analysis	47	High (76%/86%)	All	Multiple interventions	6–12 year olds: 21/39 studies reported that interventions had a sig. positive impact on PA-related factors. 13–18 years old: 5/8 studies reported that interventions had a sig positive impact on PA.
World Health Organisation (66)	Systematic review	107	Medium (50%/78%)	All	Multiple interventions	Effective interventions: School-based including a PA component in the curriculum, providing a supportive school environment, offering PA programs and ensuring parental involvement.

As one part of a multi-component approach, a range of reviews stated that interventions targeting physical education lessons are effective. Firstly, evidence shows that increasing the number of PE lessons is a key strategy to promote PA in school children (15, 72–74). Secondly, improving the quality of PE lessons can be effective (15, 75). Lonsdale et al. (75) indicated that interventions can increase time spent in moderate-to-vigorous PA by 24% in PE lessons, thus substantially influencing the total amount of PA. Third, and strongly connected to higher quality PE lessons, reviews specified teacher training and capacity building as effective strategies to promote PA in school children (15, 66, 69, 74–76).

Furthermore, several reviews provide evidence for the integration of (more) PA into the curriculum (15, 66, 69, 74, 77, 78). Evidence on integrating (PA-related) health education into the curriculum is inconclusive. While the integration of health education into the curriculum was recommended in one of the reviews (68), another review stated that results were mixed (73).

Activity breaks—the integration of short bouts of PA into organisational routine—is another effective strategy to promote PA in the school setting (15, 70, 79), and has demonstrated “modest but consistent benefits” (79).

After-school programs focused on PA and/or sports are also effective (15, 70, 80, 81). In this context, improving students' attendance rates is highly important as the effectiveness of a program depends strongly on attendance (81).

Changing the school environment also has an influence on PA in school children and is recommended in numerous reviews (63, 66, 69, 71, 73, 74). In particular, the provision of equipment for games and playgrounds (15, 67, 82) has proved effective. One review stated that the use of playground markings can increase PA in children during recess and lunchtime (67), whereas another study found inconclusive evidence (82). In one review, significant associations were found between decreased playground density and PA in children (82). Evidence on the effectiveness of interventions that only focus on environmental changes is limited (65, 83); such interventions should be integrated in multicomponent approaches (65, 83).

Numerous reviews reported positive effects when involving parents (63, 66, 71, 74, 84, 85) and families (14, 65, 70) in school-based interventions. Only one review indicated that evidence for parental involvement was inconclusive due to a lack of studies (85). One review of reviews mentioned that most but not all included records support the effectiveness of family involvement (14).

Community involvement was linked with positive outcomes in two reviews (65, 70). Surprisingly, one review stated that school-based interventions employing a community component were ineffective (70).

The involvement of pupils is another relevant factor (69), as peer support can increase PA levels in school children (63, 71). Adjusting interventions to this specific target population is necessary (15).

Promoting active transport to school is another effective strategy to promote PA among children and adolescents (15, 69, 73, 86, 87). This strategy is related to promising policies such as ensuring safe routes to school and improving urban design (72, 73).

Finally, a number of reviews in the school setting addressed whether an intervention should acknowledge several behavioural components. While Pate et al (73) didn't find any differences between interventions addressing single or multiple behavioural components, five other reviews did. The evidence shows that interventions addressing more than one health behaviour are less effective (63, 71). The integration of other components in addition to PA was considered as a “stumbling block for success” (14). This point is stressed in another review that focuses on after-school programs (80). In general, intervention effectiveness is higher when focus is placed on a specific goal compared to interventions that require broader focus (86).

### Other

Four reviews were classified as “other” as their results were not connected with one specific setting (Table 6).

**Table 6:** Reviews focusing on other settings.

**References**	**Type**	**Number of included studies**	**Quality rating (QC/AGREE)**	**Intervention type**	**Interventions included in review**	**Main findings of the authors**
**OTHER**						
Cushing et al. (89)	Systematic review with meta-analysis	58	High (68%/86%)	All	Multiple interventions across settings	Sig. effect sizes: Interventions with families, individuals, and schools (0.08), interventions with families and individuals (0.53).
Hamel et al (90)	Systematic review	14	High (71%/86%)	All, but mainly informational	Computer- and web-based interventions across settings	2/5 studies on home- or camp-based interventions reported increases in PA and/or reduction of obesity. 7/9 studies on school-based interventions reported increases in PA and/or reduction of obesity.
Hillier-Brown et al (91)	Systematic review	23	Low (54%/69%)	All	Multiple interventions across settings	Some evidence for effectiveness in reducing socio-economic inequalities in obesity-related outcomes: PA education and exercise sessions, family based education and exercise based weight loss programmes.
Kamath et al. (92)	Systematic review with meta-analysis	29	High (78%/80%)	All	Multiple interventions across settings	The meta-analysis resulted in a pooled effect size for PA of 0.12. A trend was identified in favour of multiple cognitive components (0.15; vs. one or no cognitive components, 0.00) and interventions including reinforcement (0.24; vs. no reinforcement, −0.07).

Three of these reviews analysed a broad range of health promoting interventions (89, 91, 92). As the reviews dealt with interventions not only pertaining to PA, results on efficacy are not as specific as other reviews. Overall, the reviews stated that health promotion interventions can be effective in terms of health (89), obesity prevention (92), and the reduction of socioeconomic inequalities in obesity (91). One of the reviews stated that interventions in the healthcare setting can be effective (91).

One of the reviews analysed computer- and web-based interventions in both the home and school setting (90). This intervention type can be effective, especially with regard to schools (90).

## Discussion

### Key Findings

This systematic review of reviews provided important findings regarding PA promotion for children and adolescents:

- Parents play a key role in the family and home setting. In settings such as childcare facilities and schools, evidence shows that parental involvement is an important factor for the efficacy of interventions.
- Multi-component interventions proved to be effective in the childcare setting and the school setting.
- For childcare facilities, reviews stated that interventions should be theory or model-based, include a PA programme, provide more free time, and contain teacher training.
- In schools, evidence is available for increasing the number of PE lessons, as well as improving the quality of PE lessons, teacher training and capacity building. Furthermore, findings indicate the efficacy of other intervention strategies: implementation of more PA into the curriculum; activity breaks; after-school programs; changes in the school environment; promotion of active transport; community and peer involvement.

### Research Gaps

Additionally, several gaps were identified in the evidence base. These gaps partly reflect underlying controversies regarding research paradigms utilised to study effects of PA interventions:

- Research focuses primarily on individual level interventions even though community and policy level interventions are considered to be more effective in terms of public health (61). Such focus on individual level interventions can be explained by the predominance of evidence-based medicine (92) for the evaluation of interventions. Within the paradigm of evidence-based medicine, it is of high importance to generate knowledge through randomised and controlled trials. These study designs, however, have low applicability if one wants to examine policy or environmental interventions. Due to this, a combined use of systematic reviews, meta-analysis and realist synthesis as employed in a recent analysis of family-based PA interventions (93) seems to be beneficial.
- Research is dominated by studies investigating interventions in the school setting. Interventions in the settings of family and childcare were only investigated in a limited number of reviews—even though there is a lack of knowledge regarding interventions outside of the school setting (70). One review couldn't identify a single study including effective interventions for children aged 0–5 years (74). In particular, for nations where children only spend half a day in school (for example, in Germany), school-based interventions might have less relevance for PA promotion. This is because schools have neither the capacities nor the facilities for additional interventions for PA promotion. For such nations, more knowledge is needed on PA promotion outside of the school setting.
- Research on interventions in settings other than family, childcare and school is almost non-existent. As other studies show that computer- and web-based interventions can be effective in promoting PA of children and adolescents (90), this gap in the evidence base could limit public health impact. Moreover, we could not find any evidence regarding the sport club setting. In particular, for nations which have a sport club-based system for PA in leisure-time, such knowledge could prove valuable.
- Most reviews investigated the “efficacy” of interventions with very limited information on “effectiveness” (90). This is problematic as researchers raise the question whether interventions that are successful in efficacy studies are also effective in the real world (56). From a Public Health perspective, study designs that allow for the simultaneous testing of both efficacy and effectiveness—such as pragmatic trials (93)—might be more appropriate to generate this evidence. This could allow accelerating scale-up processes of interventions. Also the RE-AIM framework can be used for analysing both efficacy and effectiveness of interventions, as recent systematic reviews of physical activity interventions for children and youth show (94, 95).

### Challenges for Physical Activity Promotion

From an international perspective, the key findings and gaps in the evidence base are associated with several challenges for PA promotion. Such challenges are mainly caused due to focus being placed on the school setting:

- Firstly, the concrete implementation of effective interventions depends on the structure of the educational system. In a study comparing the educational governance of the USA and France, the authors stated that “institutional sectors in liberal polities are often organised as complex multi-layered governance systems characterised by fragmented decision-making structures.” In state-centred polity on the other hand, “one can expect more tightly structured institutional sectors” (96). These findings affect the implementation of the results from this systematic review of reviews into practice (e.g., with regard to the number and quality of PE lessons). In Germany, the federal system's fragmented decision-making structure allows federal states to decide on school curricula (97, 98). In Hungary however, regulation on daily PE classes was implemented as a nation-wide policy in 2012/2013 (99).
- Secondly, interventions that are most needed might also be connected with overall school policy. In Germany, school lessons usually finish at 1 p.m. or 2 p.m. However, the percentage of pupils visiting all-day schools has constantly risen over the years (from 9.8% in 2002 up to 39.3% in 2015) (100). With an increasing number of pupils visiting all-day schools, the need for additional sport facilities, and changes in the school environment might be most relevant.
- For school children, the integration of sectors other than education is only investigated as part of a multi-component approach. The necessity of focusing on the family setting is stressed by findings on the importance of parental involvement. For the promotion of active transport to school, the influence of urban planning requires further investigation.
- The question of how to finance interventions in the education sector was not even raised in the reviews, even though the lack of public funding and resources is perceived as a barrier for childhood obesity prevention by two thirds of stakeholders in Europe (101). Intersectoral partnerships might be a promising approach to finance programs with combined resources.

### National Recommendations for Physical Activity Promotion

Based on this comprehensive and up-to-date cross-sectoral review, national recommendations for physical activity promotion were developed in Germany. These recommendations aim to have an impact on public health by offering scientific orientation for experts and stakeholders. Alongside the efficacy of interventions, the national recommendations also consider aspects regarding PA promotion in children and adolescents (102):

- Effectiveness: In terms of public health, did the intervention prove to be effective on a large scale?
- Health equity: Is the intervention able to address and reduce health inequalities?
- Cost effectiveness: Does the intervention demonstrate a good relation between costs and the expected benefits?
- Quality criteria: Which criteria need to be considered to ensure the successful implementation of an intervention?

Considering these aspects is highly beneficial for bridging the gap between evidence and practice. For decision makers, effectiveness and health equity are important criteria, as well as cost effectiveness. Furthermore, quality criteria are important for practitioners, and decision makers: For example, evidence shows that the involvement of all relevant stakeholders—children, families, teaching staff and management—in the planning of an intervention increases the likelihood of its success (102–108).

In order to improve the evidence base, future research on PA promotion for children and adolescents should focus on the above-mentioned aspects (109).

## Author Contributions

AR acquired the funding, administrated the project, developed concept and methodology of this study and analysed the data. SM supported both literature search and data analysis and wrote the original manuscript draft. KA-O oversaw the literature search and data analysis and supported the development of the original draft. UU-R performed data analyses. LG contributed to manuscript preparation through the revision of numerous drafts. IB supported the literature search. GG developed the quality assessment tool and assessed the quality of reviews. All authors critically reviewed and edited the manuscript.

### Conflict of Interest Statement

The authors declare that the research was conducted in the absence of any commercial or financial relationships that could be construed as a potential conflict of interest.

## Abbreviations

PA: Physical activity
WHO: World Health Organisation.
